# Indoleamine 2,3-Dioxygenase Is Not a Pivotal Regulator Responsible for Suppressing Allergic Airway Inflammation through Adipose-Derived Stem Cells

**DOI:** 10.1371/journal.pone.0165661

**Published:** 2016-11-03

**Authors:** Kyu-Sup Cho, Mi-Kyung Park, Sue-Jean Mun, Hee-Young Park, Hak-Sun Yu, Hwan-Jung Roh

**Affiliations:** 1 Department of Otorhinolaryngology and Biomedical Research Institute, Pusan National University Hospital, Busan, Republic of Korea; 2 Department of Parasitology, Pusan National University School of Medicine, Yangsan, Republic of Korea; 3 Department of Otorhinolaryngology and Research Institute for Convergence of Biomedical Science and Technology, Pusan National University Yangsan Hospital, Yangsan, Republic of Korea; Mie University Graduate School of Medicine, JAPAN

## Abstract

**Background:**

Although indoleamine 2,3-dioxygenase (IDO)-mediated immune suppression of mesenchymal stem cells (MSCs) has been revealed in septic and tumor microenvironments, the role of IDO in suppressing allergic airway inflammation by MSCs is not well documented. We evaluated the effects of adipose-derived stem cells (ASCs) on allergic inflammation in IDO-knockout (KO) asthmatic mice or asthmatic mice treated with ASCs derived from IDO-KO mice.

**Methods and Findings:**

ASCs were injected intravenously in wild-type (WT) and IDO-KO asthmatic mice. Furthermore, asthmatic mice were injected with ASCs derived from IDO-KO mice. We investigated the immunomodulatory effects of ASCs between WT and IDO-KO mice or IDO-KO ASCs in asthmatic mice. In asthmatic mice, ASCs significantly reduced airway hyperresponsiveness, the number of total inflammatory cells and eosinophils in bronchoalveolar lavage fluid (BALF), eosinophilic inflammation, goblet hyperplasia, and serum concentrations of total and allergen-specific IgE and IgG1. ASCs significantly inhibited Th2 cytokines, such as interleukin (IL)-4, IL-5, and IL-13, and enhanced Th1 cytokine (interferon-γ) and regulatory cytokines (IL-10, TGF-β) in BALF and lung draining lymph nodes (LLNs). ASCs led to significant increases in regulatory T-cells (Tregs) and IL-10^+^ T cell populations in LLNs. However, the immunosuppressive effects of ASCs did not significantly differ between WT and IDO-KO mice. Moreover, ASCs derived from IDO-KO mice showed immunosuppressive effects in allergic airway inflammation.

**Conclusions:**

IDO did not play a pivotal role in the suppression of allergic airway inflammation through ASCs, suggesting that it is not the major regulator responsible for suppressing allergic airway inflammation.

## Introduction

Allergic rhinitis and asthma are characterized by Th2-skewed eosinophilic inflammation, mucus hypersecretion, and airway hyperresponsiveness [1]. The excessive activation of Th2 cells by insufficient suppression of regulatory T-cells (Tregs) is thought to play a major role in the initiation and development of allergic airway diseases [2–4]. Several studies have shown that mesenchymal stem cells (MSCs) provide a significant reduction in allergic airway inflammation and improve lung function [5–11]. Although the immunomodulatory mechanism of MSCs in allergic airway diseases remains to be elucidated, it has been suggested that upregulation of Tregs and increases in several soluble factors, such as prostaglandin E2 (PGE2), transforming growth factor-β (TGF-β), and interleukin (IL)-10 play critical roles in alleviating allergic airway inflammation through MSCs [12–15]. Furthermore, MSCs derived from adipose tissue (ASCs) significantly increase serum levels of PGE2 and the expression of TGF-β and indoleamine 2, 3-dioxygenase (IDO) in lung tissue responsible for the increase in Tregs in asthmatic mice [12].

IDO is an intracellular heme-containing enzyme that catalyzes the initial rate-limiting step in tryptophan degradation along the kynurenine pathway [16]. It is a pivotal regulator of the immune response and an important player in tumor immunosurveillance [17–19]. Induction of IDO results in the depletion of cellular tryptophan levels and the production of kynurenines that inhibit T cell activation and induce the proliferation of immunosuppressive Tregs [20,21]. Furthermore, IDO-mediated tryptophan catabolism is a novel T-cell inhibitory effector mechanism in human and mice MSCs [20,22]. Although IDO-mediated immune suppression by MSCs has been revealed in septic and tumor microenvironments [22–24], the role of IDO in suppression of allergic airway inflammation by MSCs is not well documented.

In this study, we investigated whether IDO contributes to the immunomodulatory effects of MSCs in asthmatic mice by evaluating the effects of MSCs on allergic inflammation in IDO-knockout (KO) mice or mice treated with ASCs derived from IDO-KO mice.

## Materials and Methods

### Animals

Five-week-old female wild-type (WT) mice and IDO-KO mice with a C57BL/6 background were obtained from The Jackson Laboratory (Bar Harbor, ME; http://www.jax.org) and bred in a specific-pathogen-free animal facility. The animal study protocol was approved by the Institutional Animal Care and Use Committee of the Pusan National University School of Medicine.

### Isolation and culture of ASCs

Among the MSCs, ASCs were used because of their abundance, relative ease of harvesting, and high proliferation potential. Adipose tissue was obtained from the abdominal fat of WT or IDO-KO C57BL/6 mice, washed extensively with equal volumes of phosphate-buffered saline (PBS), and digested with 0.075% collagenase type I (Sigma, St. Louis, MO) at 37°C for 30 min. Enzyme activity was neutralized using α-modified Eagle’s medium (α-MEM) containing 10% fetal bovine serum (FBS) followed by centrifugation at 1,200 × g for 10 min to obtain a pellet. The pellet was filtered through a 100 μm nylon mesh to remove cellular debris and then incubated overnight at 37°C with 5% CO_2_ in control medium (α-MEM, 10% FBS, 100 unit/mL penicillin, 100 μg/mL streptomycin). Following incubation, the plates were washed extensively with PBS to remove residual non-adherent red blood cells. The resulting cell population was maintained at 37°C with 5% CO_2_ in control medium. One week later, after the monolayer of adherent T-cells had reached confluence, cells were trypsinized (0.05% trypsin-EDTA; Sigma), resuspended in α-MEM containing 10% FBS, and subcultured at a concentration of 2,000 cells/cm^3^. For the experiments, third- or fourth-passage ASCs were used.

Flow cytometric analysis was used to characterize the ASC phenotype. At least 50,000 cells (in 100 μL PBS, 0.5% bovine serum albumin [BSA], 2 mmol/L EDTA) were incubated with fluorescein isothiocyanate-labeled monoclonal antibodies (Abs) against mouse stem cell antigen-1 (Sca-1), CD44, CD90, CD45, CD117, and CD11b (BD Biosciences Clontech, Palo Alto, CA) or with the respective isotype control. After washing, labeled cells were analyzed by flow cytometry using a FACSCalibur flow cytometer and Cell Quest Pro software (BD Biosciences, San Diego, CA). The expression percentage of each marker of ASCs was determined by the percentage of positive events, as determined compared to the isotype-matched negative control.

ACSs were analyzed for their capacity to differentiate into adipogenic, osteogenic, and chondrogenic lineages, as described previously [25]. For adipogenic and osteogenic differentiation, cells were seeded in 6-well plates at a density of 20,000 cells/cm^2^ and treated for 3 weeks with adipogenic and osteogenic media. Adipogenic and osteogenic differentiation was assessed using oil red O staining, as an indicator of intracellular lipid accumulation, and alizarin red S staining, as an indicator of extracellular matrix calcification. Chondrogenic differentiation was induced using the micromass culture technique. Briefly, 10 mL a concentrated ASC suspension (3 × 10^5^ cells/mL) were plated in the center of each well and treated for 3 weeks with chondrogenic medium. Chondrogenesis was confirmed by immunohistochemistry.

### Mouse model of allergic airway inflammation

**Fig 1:**
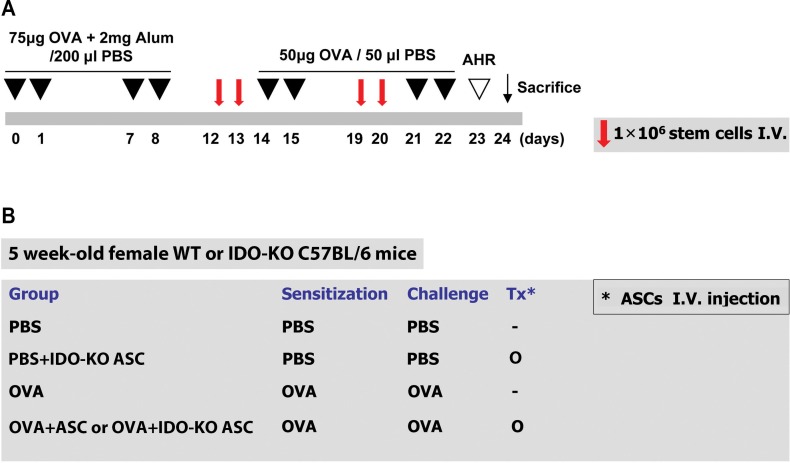
The experimental protocol. (A) Mice were sensitized on days 0, 1, 7, and 8 by intraperitoneal injection of ovalbumin (OVA) and challenged intranasally on days 14, 15, 21, and 22. Purified adipose-derived stem cells (ASCs; 1 × 10^6^) derived from wild-type (WT) or indoleamine 2, 3-dioxygenase (IDO)-knockout (KO) mice were injected via the tail vein on days 12, 13, 19, and 20. (B) Mice were divided into three or four treatment groups.

A mouse model of allergic airway inflammation was induced as previously reported with minor modifications [12,26]. Briefly, mice were sensitized by intraperitoneal injection of 75 μg ovalbumin (OVA, Sigma, St. Louis, MO; http://www.sigmaaldrich.com) with 2 mg aluminum hydroxide (Sigma) in 200 μL PBS on days 0, 1, 7, and 8. On days 14, 15, 21, and 22 after the initial sensitization, the mice were challenged intranasally with 50 μg OVA in 50 μL PBS. The mice were sacrificed via CO_2_ inhalation on day 24 (Fig 1A).

### Intravenous transplantation of ASCs or IDO-KO ASCs

ASCs were washed with PBS and suspended in PBS at a concentration of 1 × 10^7^ cells/mL. To evaluate the effects of ASCs or ASCs derived from IDO-KO mice, 0.1 mL purified ASCs were injected using a 26-gauge needle via the tail vein of WT and IDO-KO asthmatic mice once a day on days 12, 13, 19, and 20, while IDO-KO ASCs were injected in WT asthmatic mice.

Mice were divided into three or four groups of five mice per group: (a) PBS group sensitized, pretreated, and challenged with PBS; (b) PBS+IDO-KO ASC group sensitized and challenged with PBS, but pretreated with IDO-KO ASCs; (c) OVA group sensitized with OVA, pretreated with PBS, and then challenged with OVA; (d) OVA+ASC group or OVA+IDO-KO ASC group sensitized with OVA, pretreated with ASCs or IDO-KO ASCs, and then challenged with OVA. These experiments were performed four times (Fig 1B).

### Measurement of methacholine airway hyperresponsiveness

Twenty-four hours after the last challenge, airway hyperresponsiveness (AHR) was assessed in conscious, unrestrained mice using noninvasive whole-body plethysmography (Allmedicus, Seoul, Republic of Korea), as described previously [27]. Briefly, mice were placed in the plethysmography chamber and exposed to increasing concentrations of aerosolized methacholine at 0, 12.5, 25, and 50 mg/mL for 10 min. Enhanced pause (Penh) was calculated automatically based on the mean pressure generated in the plethysmography chamber during inspiration and expiration combined with the time of each phase. Then the Penh values calculated during each 3 min interval were averaged.

### Differential cell counts in bronchoalveolar lavage fluid

To obtain bronchoalveolar lavage fluid (BALF), the tracheas of anesthetized mice were exposed and cut just below the larynx. A polyurethane flexible tube (0.4 mm in outer diameter, 4 cm in length, and attached to a blunt 24-gauge needle [Boin Medical Co., Seoul, Republic of Korea]) was placed into the trachea, after which the lung was lavaged once with 800 mL warm sterile PBS. The BALF samples were centrifuged for 5 min at 1,500 rpm at 4°C. Then the supernatants were decanted and frozen immediately at -70°C. Cell pellets were resuspended and washed twice in PBS. The total cell numbers were counted using a hemocytometer. BALF cell smears were prepared using a cytospin apparatus and stained with Diff-Quik solution (Sysmex Co., Kobe, Japan) to determine the differential cell counts in accordance with conventional morphological criteria. At least 500 cells per slide were evaluated to obtain the differential leukocyte counts.

### Lung histology and inflammation scoring

Lung tissues were removed after lavage, fixed in 10% neutral formalin for 36 h, and embedded in paraffin. Thin sections of the embedded tissues were stained with hematoxylin and eosin (H&E) and periodic acid-Schiff (PAS) to identify eosinophils and count mucin-secreting cells, respectively. Lung inflammation was assessed by the degree of peribronchial and perivascular inflammation, which was evaluated on a subjective scale of 0–4, as described previously [28,29], using the following inflammatory parameters: 0, no inflammation detectable; 1, occasional cuffing with inflammatory cells; 2, most bronchi or vessels surrounded by a depth of one to three cells; 3, most bronchi or vessels surrounded by a depth of four to five cells; 4, most bronchi or vessels surrounded by a depth of more than five cells. To quantify goblet cell hyperplasia, the percentage of PAS-positive cells in epithelial areas was determined from 8–10 tissue sections per mouse.

### Measurement of serum levels of immunoglobulin and PGE2

At 48 h after the last OVA challenge, serum was collected from mice via cardiac puncture. The levels of total and OVA-specific immunoglobulins (Ig E, IgG1, IgG2a) and PGE2 were determined by enzyme-linked immunosorbent assay (ELISA) in accordance with the manufacturer’s instructions (R&D Systems, Minneapolis, MN). Absorbance at 450 nm was measured using an ELISA plate reader (Molecular Devices, Sunnyvale, CA).

### Expression of cytokines in BALF and lung draining lymph nodes

Lung draining lymph nodes (LLNs) were obtained from between the trachea and both lung lobes. The obtained LLNs were treated with ACK hypotonic lysis buffer (0.15 M NH_4_Cl, 1mM KHCO_3_, 0.1mM Na_2_-EDTA, pH 7.2–7.4) for 2 min at room temperature to lyse red blood cells (RBCs). After the RBCs were lysed, the remaining cells were filtered using a 100 μm mesh (Small Parts Inc., Miramar, FL) and 10^6^ cells/mL were plated in 48-well plates coated with 0.5 μg/mL CD3 Ab (BD Biosciences) in RPMI 1640 with 10% fetal bovine serum (FBS) and penicillin/streptomycin. Plated cells were incubated for 72 h at 37°C with 5% CO_2_. After stimulation, the concentrations of mouse IL-4, IL-5, IL-10, IL-13, interferon (IFN)-γ, and TGF-β in BALF and in stimulated LLN supernatant were examined using commercially available ELISA kits following the manufacturer’s instructions (eBioscience, San Diego, CA). The absorbance of the final reactant was determined at 450 nm using an ELISA plate reader (Molecular Devices).

### Quantitative real-time PCR for IDO and TGF-β

RNA was extracted from the lungs using 1 mL QIAzol (Qiagen science, Valencia, CA) and following the manufacturer’s protocols, transcribing 2 μg of RNA using moloney murine leukemia virus (M-MLV) reverse transcriptase (Promega, Madison, WI). Indoleamine 2,3-dioxygenase (IDO) (forward, 5’-GATGAAGAAGTGGGCTTTGC-3’; reverse, 5’-TCCAGTTTGCCAAGACACAG-3’) and TGF-β (forward, 5′-CTACCTTTCCTTGGGAGACC-3′; reverse, CGGGAGTGGGAGCAGAA-3′) RNA levels were quantified, relative to the housekeeping gene GAPDH, using iCycler^TM^ (Bio-Rad laboratories Inc., Hercules, CA) real-time PCR machines following the manufacturer’s instructions. Then the relative expression of each gene was calculated as the ratio to the housekeeping gene using the gene-x program (Bio-Rad laboratories Inc.).

### Determination of Tregs and intracellular cytokine staining

To evaluate the recruitment of Th1, Th2, and Tregs induced by ASCs treatment, LLN cells from OVA-induced asthmatic mice and ASC-treated asthmatic mice were cultured in anti-CD3-coated plates for 6 h. To evaluate CD4^+^CD25^+^Foxp3^+^ (Tregs) and IL-10^+^/CD4+ T-cells, cells were stained with anti-CD4-FITC (0.5 mg/mL) and anti-CD25-APC (0.2 mg/mL) following the manufacturer’s recommendations (eBiosciences, San Diego, CA). After surface staining, cells were permeabilized using the Cytofix/Cytoperm Kit (BD Biosciences). After permeabilization, cells were stained with anti-Foxp3-PE-cy7 or anti-IL-10-PE (eBiosciences).

To assess the Th1 and Th2 cell populations, LLN cells were stained with anti-CD4-FITC Ab. After surface staining, CD4+ T-cells were stained with intracellular anti-IFN-γ-PE-cy7 (eBiosciences) and anti-IL-4-PE (eBiosciences) Abs. Fluorescence was measured using a FACS CantoII cytometer (BD Biosciences) equipped with Canto software (BD Biosciences).

### Statistical analysis

All experiments were repeated a minimum of three times. Data are expressed as means ± standard error of the mean (SEM). Statistical significance was assessed by Student’s *t* test or one-way analysis of variance (ANOVA) using the SPSS software package version 18.0 (SPSS Inc., Chicago, IL). A p value < 0.05 was considered statistically significant.

## Results

### Characterization of ASC immunophenotype and differentiation

The cultured ASCs from adipose tissue of WT and IDO-KO C57BL/6 mice were negative for the cell surface markers CD45, CD117, and CD11b, but positive for Sca-1, CD44, and CD90. These putative ASCs had a spindle-shaped fibroblast-like appearance, similar to previously reported adipose tissue and bone marrow-derived MSCs. ASCs had the ability to differentiate into adipogenic, osteogenic, and chondrogenic lineages after culture in the appropriate conditions (S1 Fig).

### AHR and inflammatory cells in BALF

**Fig 2:**
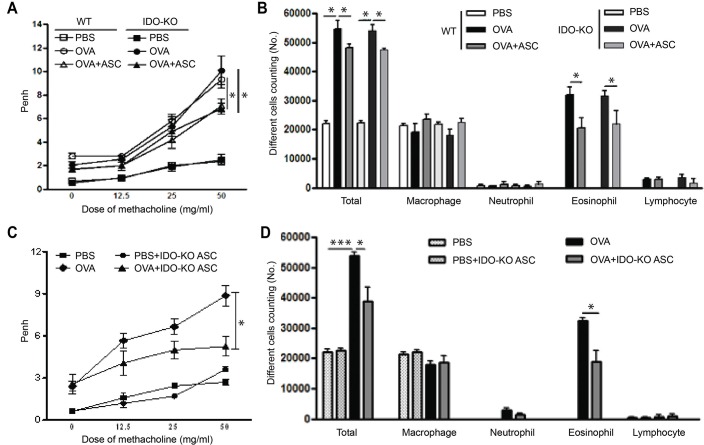
Effects of adipose-derived stem cells (ASCs) on airway hyperresponsiveness (AHR) and inflammatory cells in the bronchoalveolar lavage fluid (BALF). ASCs or ASCs derived from indoleamine 2, 3-dioxygenase (IDO)-knockout (KO) mice significantly decreased AHR (A, C) and the number of total inflammatory cells and eosinophils (B, D) in wild-type (WT) and IDO-KO asthmatic mice. Data are expressed as the mean ± SEM of four independent experiments performed in triplicate. _*_ *p*<0.05, _***_ *p*≤0.001.

Penh in WT and IDO-KO asthmatic mice increased with methacholine concentration, and ASC treatment significantly decreased AHR in WT and IDO-KO asthmatic mice (*p* = 0.043 and *p* = 0.048, respectively) (Fig 2A). Furthermore, treatment with ASCs derived from IDO-KO mice significantly decreased AHR in the OVA+IDO-KO ASC group (*p* = 0.020) (Fig 2C).

The numbers of total inflammatory cells and eosinophils were significantly increased in BALF from the OVA group compared to the PBS group. However, ASC treatment significantly decreased the numbers of total inflammatory cells (*p* = 0.034 and *p* = 0.030, respectively) and eosinophils (*p* = 0.007 and *p* = 0.008, respectively) in WT and IDO-KO asthmatic mice (Fig 2B). Moreover, treatment with ASCs derived from IDO-KO mice significantly decreased the numbers of total inflammatory cells and eosinophils in asthmatic mice (*p* = 0.037 and *p* = 0.018, respectively) (Fig 2D).

### Lung inflammation and goblet cell hyperplasia

**Fig 3:**
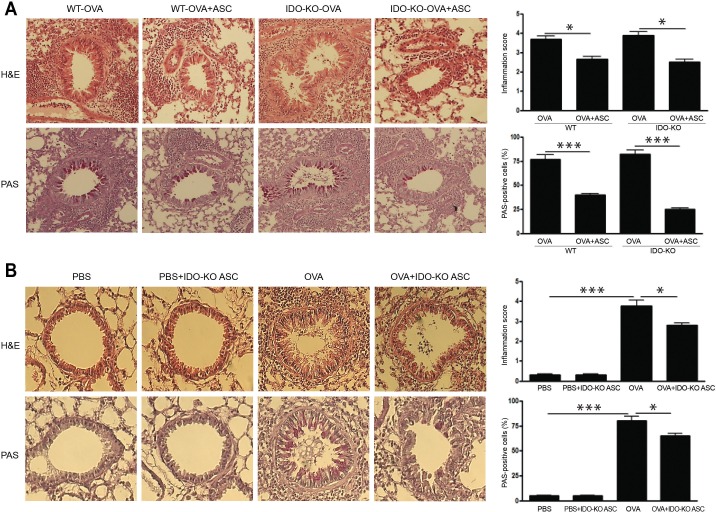
Effects of adipose-derived stem cells (ASCs) on lung inflammation and goblet cell hyperplasia. ASCs or ASCs derived from indoleamine 2, 3-dioxygenase (IDO)-knockout (KO) mice decreased the infiltration of eosinophils and PAS-positive cells around the airways and blood vessels in wild-type (WT) and IDO-KO asthmatic mice (H&E, PAS ×200). ASCs (A) or IDO-KO ASC (B) treatment significantly reduced the inflammation score and PAS-positive cells. Data are expressed as the mean ± SEM of four independent experiments performed in triplicate. _*_ *p*<0.05, _***_ *p*≤0.001.

No obvious infiltration of inflammatory cells was found in the PBS and PBS+IDO-KO ASC groups, but a greater number of eosinophils in the peribronchial and perivascular areas were seen in WT and IDO-KO asthmatic mice. Goblet cell hyperplasia was demonstrated by the increased number and size of goblet cells following PAS staining within the respiratory epithelium in WT and IDO-KO asthmatic mice. However, no obvious infiltration of inflammatory cells or goblet cell hyperplasia was found in asthmatic mice treated with ASCs or IDO-KO ASCs. Furthermore, ASC administration led to a significant reduction in the inflammation score (*p* = 0.009 and *p* = 0.007, respectively) and PAS-positive cells (all *p<*0.001) in WT and IDO-KO asthmatic mice (Fig 3A).

In addition, IDO-KO ASC treatment induced a significant reduction in the number of eosinophils (*p* = 0.047) and goblet cell hyperplasia (*p* = 0.043) in asthmatic mice (Fig 3B).

### Serum levels of total and OVA-specific IgE, IgG1, and IgG2a

**Fig 4:**
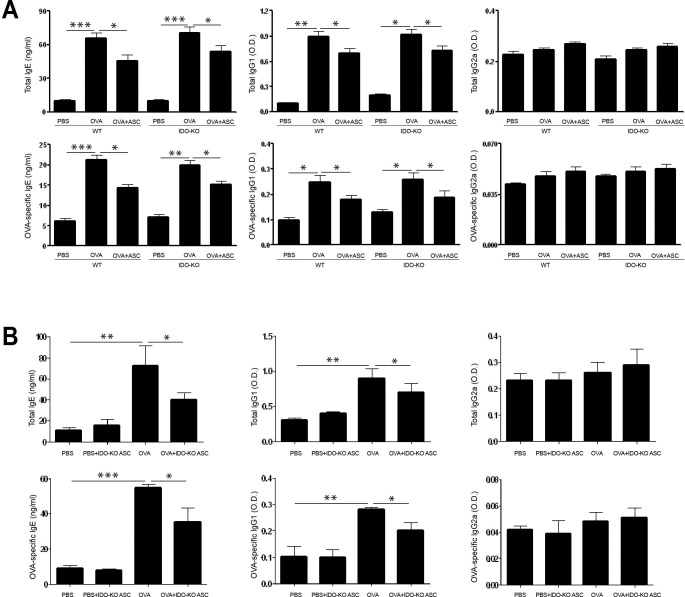
Effect of adipose-derived stem cells (ASCs) on serum levels of immunoglobulin. Systemic administration of ASCs (A) or ASCs derived from indoleamine 2, 3-dioxygenase (IDO)-knockout (KO) mice (B) resulted in a significant decrease in total IgE, total IgG1, OVA-specific IgE, and OVA-specific IgG1 in WT and IDO-KO asthmatic mice. Data are expressed as the mean ± SEM of four independent experiments performed in triplicate. _*_ *p*<0.05, _**_ *p*≤0.005, _***_ *p*≤0.001.

Levels of total and OVA-specific IgE and IgG1 were significantly higher in the OVA group than in the PBS group of WT and IDO-KO asthmatic mice. However, systemic administration of ASCs significantly decreased total IgE (*p* = 0.038 and *p* = 0.026, respectively), total IgG1 (*p* = 0.039 and *p* = 0.037, respectively), OVA-specific IgE (*p* = 0.033 and *p* = 0.048, respectively), and OVA-specific IgG1 (*p* = 0.025 and *p* = 0.037, respectively) in WT and IDO-KO asthmatic mice (Fig 4A).

ASCs derived from IDO-KO mice induced a significant decrease in total IgE and IgG1 (*p* = 0.049 and *p* = 0.037, respectively) and OVA-specific IgE and IgG1 levels (*p* = 0.016 and *p* = 0.012, respectively) in the OVA+IDO-KO ASC group. There were no significant differences in total and OVA-specific IgG2a levels among the groups (Fig 4B).

### Cytokine profiles in BALF and LLN

**Fig 5:**
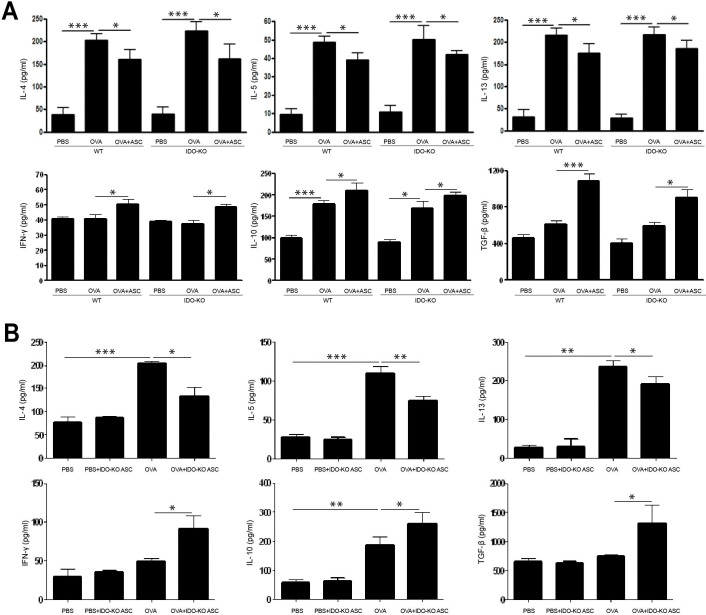
Effect of adipose-derived stem cells (ASCs) on cytokine levels in bronchoalveolar lavage fluid. IL-4, IL-5, and IL-13 levels were significantly higher in the OVA group than PBS group. ASCs (A) or ASCs derived from indoleamine 2, 3-dioxygenase (IDO)-knockout (KO) mice (B) treatment significantly decreased IL-4, IL-5, and IL-13, but increased IFN-γ, IL-10, and TGF-β in WT and IDO-KO asthmatic mice. Data are expressed as the mean ± SEM of four independent experiments performed in triplicate. _*_ *p*<0.05, _**_ *p*≤0.005, _***_ *p*≤0.001.

**Fig 6:**
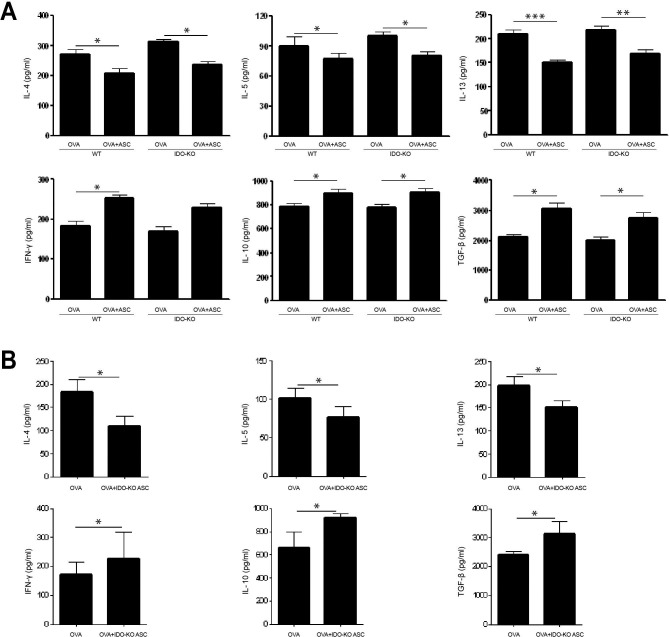
Effect of adipose-derived stem cells (ASCs) on cytokine levels in the lung draining lymph nodes. ASCs (A) or ASCs derived from indoleamine 2, 3-dioxygenase (IDO)-knockout (KO) mice (B) treatment significantly decreased IL-4, IL-5, and IL-13 levels, but increased IFN-γ, IL-10, and TGF-β levels in WT and IDO-KO asthmatic mice. Data are expressed as the mean ± SEM of four independent experiments performed in triplicate. _*_ *p*<0.05, _**_ *p*≤0.005, _***_ *p*≤0.001.

OVA-challenged mice showed significantly increased levels of IL-4, IL-5, and IL-13 in BALF. However, ASC treatment significantly decreased IL-4, IL-5, and IL-13 in BALF (*p* = 0.023 and *p* = 0.021, *p* = 0.013 and *p* = 0.034, and *p* = 0.030 and *p* = 0.048, respectively) and LLN (*p* = 0.008 and *p* = 0.008, *p* = 0.032 and *p* = 0.029, and *p*<0.001 and *p* = 0.003, respectively) of WT and IDO-KO asthmatic mice. In contrast, ASC treatment significantly increased IFN-γ, IL-10, and TGF-β levels in BALF (*p* = 0.043 and *p* = 0.037, *p* = 0.049 and *p* = 0.041, and *p<*0.001 and *p* = 0.013, respectively) and LLN (*p* = 0.007 and *p* = 0.037, *p* = 0.049 and *p* = 0.039, and *p* = 0.039 and *p* = 0.030, respectively) of WT and IDO-KO asthmatic mice (Figs 5A and 6A).

ASCs derived from IDO-KO mice led to a significant decrease in IL-4 (*p* = 0.034 and *p* = 0.019, respectively), IL-5 (*p* = 0.005 and *p* = 0.040, respectively), and IL-13 (*p* = 0.040 and *p* = 0.032, respectively) in BALF and LLN from the OVA+IDO-KO ASC group. However, IDO-KO ASC treatment significantly increased IFN-γ (*p* = 0.028 and *p* = 0.037, respectively), IL-10 (*p* = 0.049 and *p* = 0.038, respectively), and TGF-β (*p* = 0.035 and *p* = 0.047, respectively) in BALF and LLN from the OVA+IDO-KO ASC group (Figs 5B and 6B).

### Expression of IDO, TGF-β, and PGE2

**Fig 7:**
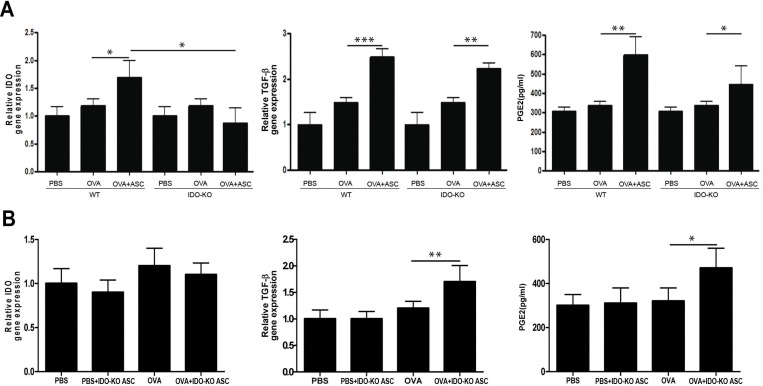
Effect of adipose-derived stem cells (ASCs) on the expression of IDO, TGF-β, and PGE2. ASCs (A) or ASCs derived from indoleamine 2, 3-dioxygenase (IDO)-knockout (KO) mice (B) treatment significantly increased TGF-β expression in lung tissue and PGE2 levels in the serum of asthmatic mice. However, IDO expression in lung tissue was increased in the OVA+ASC group of WT asthmatic mice, but not in the OVA+ASC group of IDO-KO asthmatic mice and OVA+IDO-KO ASC group. Data are expressed as the mean ± SEM of four independent experiments performed in triplicate. _*_ *p*<0.05, _**_ *p*≤0.005, _***_ *p*≤0.001.

The gene expression levels of TGF-β in lung tissue and PGE2 levels in serum were significantly increased in the OVA+ASC group of WT (*p*<0.001 and *p* = 0.003, respectively) and IDO-KO (*p* = 0.004 and *p* = 0.032, respectively) asthmatic mice compared to the OVA group (Fig 7A). ASCs derived from IDO-KO mice significantly increased the gene expression of TGF-β and serum levels of PGE2 in the OVA+IDO-KO ASC group (*p* = 0.004 and *p* = 0.024, respectively) (Fig 7B). Gene expression levels of IDO were increased in the OVA+ASC group of WT asthmatic mice, but not in the OVA+ASC group of IDO-KO asthmatic mice and OVA+IDO-KO ASC group (Fig 7). Furthermore, there were no significant differences in the PGE2 levels of IDO-KO mice or mice treated with IDO-KO ASCs compared to WT.

### T-cell populations in LLN

**Fig 8:**
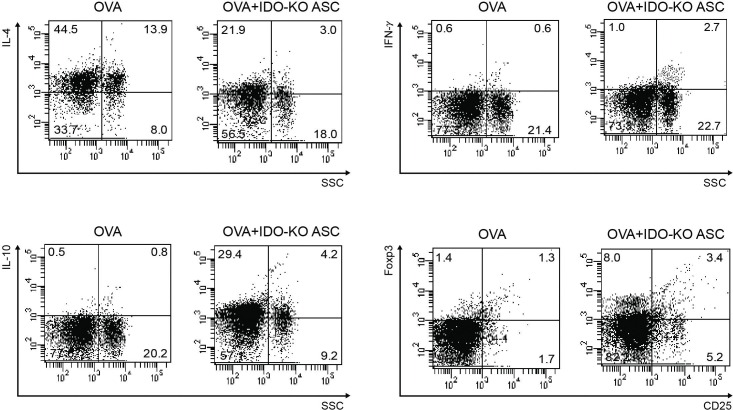
Effects of adipose-derived stem cells (ASCs) on T-cells in the lung draining lymph nodes. The CD4^+^ T-cells were initially gated and the percentage of IL-4^+^, IFN-γ^+^, IL-10^+^, and CD25^+^ Foxp3^+^ T-cells subsequently analyzed. When treating asthmatic mice with ASCs derived from indoleamine 2, 3-dioxygenase (IDO)-knockout (KO) mice, the IL-4^+^ T-cell population decreased, but the IFN-γ^+^, IL-10^+^, and Foxp3^+^CD25^+^ T-cell populations increased.

The populations of CD4^+^CD25^+^Foxp3^+^ T-cells and CD4^+^IL-10^+^ T-cells were markedly increased by administration of IDO-KO ASCs in asthmatic mice. In the OVA+IDO-KO ASC group, CD4^+^IL-4^+^ T-cells were significantly decreased and CD4^+^IFN-γ^+^ T-cells were significantly increased compared to the OVA group (Fig 8).

## Discussion

The immunomodulatory function of MSCs has led to increasing interest in them as promising candidates for the treatment of allergic airway diseases. Although the immunomodulatory mechanism of MSCs in allergic airway diseases is not completely understood, the induction and expansion of Tregs may play an important role in the suppression of allergic airway inflammation by MSCs [12–15]. The expansion of Tregs by MSCs involves not only direct contact between MSCs and CD4^+^ T-cells, but also the induction of several mediators, such as PGE2, TGF-β, and IDO [30]. However, the potential role of these possible mediators in the immune modulation of allergic airway inflammation by MSCs remains to be elucidated.

IDO is widely expressed in a variety of cell types, including B cells, macrophages, eosinophils, dendritic cells, endothelial cells, and many types of tumor cells [31–34]. IDO expression plays a critical role in the regulation of T-cell-mediated immune responses by providing a tryptophan-deficient microenvironment and limiting the accumulation of toxic metabolites of tryptophan [19]. Furthermore, the accumulation and secretion of immunosuppressive tryptophan catabolites leads to induction of T-cell anergy, apoptosis, and increased proliferation of Tregs [35,36]. This immunomodulatory function of IDO plays an essential role in a variety of pathophysiological processes, including antimicrobial and antitumor defense, immune-regulation, antioxidant activity, suppression of autoimmunity, and transplanted tissue rejection [37]. Given that the lung has at least two resident cell types that constitutively express IDO protein (endothelial cells and lung myeloid dendritic cells [DCs]), and two other types that can be recruited in larger numbers during infections or allergic inflammation (plasmacytoid DCs and eosinophils), it appears that the function of IDO is stimulatory or inhibitory depending on the target cell, the stimulus, and specific model [38]. Several recent studies have demonstrated that the IDO pathway contributes substantially to the control of allergic inflammation [39,40]. IDO activation during systemic allergen immunotherapy leads to tolerance induction regarding allergic airway inflammation [41,42]. To the best of our knowledge, this is the first study to investigate the potential role of IDO in the modulation of allergic airway inflammation by MSCs through the use of IDO-KO mice or mice treated with ASCs derived from IDO-KO mice.

MSCs may have immunosuppressive effects through the production of inducible IDO. Upregulation of IDO expression using IFN-γ within MSCs reduces inflammatory conditions [43–45]. IFN-γ induces MSCs to express the IDO protein and exhibit functional activity of IDO, leading to suppressed lymphocyte proliferation [11]. IDO expression by MSCs suppresses immune responses in Th1-mediated diseases, such as inflammatory bowel diseases, malignant tumor diseases, experimental autoimmune encephalomyelitis, collagen-induced arthritis, multiple sclerosis, and allograft rejection [24,46–51]. The use of competitive inhibitors of IDO reduces the immunosuppressive effects of MSCs in alloantigen-activated CD4+ T lymphocytes [52]. Furthermore, IDO is involved in decreasing the proliferation and cytotoxic activity of natural killer cells activated by IL-2 in the presence of MSCs and also in inhibiting the maturation and functional activity of dendritic cells [53,54].

Currently, the bulk of the literature on suppression of immune responses by IDO in the presence of MSCs is related to Th1-mediated immune responses. For Th2-mediated experimental asthma, this study showed that the immunosuppressive effects of ASCs did not significantly differ between WT and IDO-KO mice. Furthermore, in mice treated with IDO-KO ASCs, treated mice showed reductions in AHR, inflammatory cells in BALF, lung inflammation, asthma-specific cytokines in BALF and LLN, and serum levels of Th2 immunoglobulins. Importantly IDO-KO ASCs increased the induction and expansion of Tregs. In recent studies, blocking PGE2 and TGF-β eliminated the beneficial effects of ASC treatment in asthmatic mice and resulted in decreased induction of Tregs expansion by MSCs [15,55–57], suggesting that MSC-derived PGE2 and TGF-β may be the major soluble factors responsible for suppressing allergic airway inflammation. In contrast to the blocking of PGE2 and TGF-β activity, the genetic loss of IDO had no significant impact on the induction of Tregs expansion by ASCs. These findings indicate that IDO does not play a pivotal role in the suppression of allergic airway inflammation by ASCs, although IDO-mediated immune suppression of MSCs has been revealed in septic and tumor microenvironments [22–24].

These contradictory results related to IDO are difficult to resolve, but may be due to differences in the surrounding microenvironment. The present study showed that the immunosuppressive properties of IDO by MSCs could be affected by specific disease-related tissue microenvironments. Although IFN-γ is a key Th1 cytokine and a potent activator of IDO expression, Th2 cytokines such as IL-4 and IL-13 inhibit the expression of IDO [58]. T-cell suppression by ASCs appears to depend, in part, on cross-talk between T-cells and ASCs, leading to the production of IFN-γ with increased IDO expression, which in turn inhibits the proliferation of activated T-cells. Furthermore, IDO expression is not constitutive, but depends on immunologic signals mainly exerted by IFN-γ. Based on the immune-modulating effects of IDO in Th1-mediated responses, IDO modulates the host response depending on the inflammatory environment. To improve these findings, it is necessary to evaluate the induction of IDO in ASCs stimulated by various concentrations of IFN-γ *in vitro* or ASCs isolated from transgenic mice expressing green fluorescent protein.

In conclusion, the genetic loss of IDO had no significant impact on the suppression of allergic airway inflammation by ASCs, suggesting that IDO is not the major mediator responsible for suppressing allergic airway inflammation.

## Supporting Information

S1 Fig Characteristics of adipose-derived stem cells (ASCs). ASCs show characteristics of mesenchymal stem cells in the immunophenotypic analysis (A), fibroblast-like morphology (B), adipogenesis (C), osteogenesis (D), chondrogenesis (E) (original magnification
(PDF)

## References

[1] Bousquet J, Khaltaev N, Cruz AA, Denburg J, Fokkens WJ, Togias A, et al Allergic Rhinitis and its Impact on Asthma (ARIA) 2008 update (in collaboration with the World Health Organization, GA(2)Len and Allergen) Allergy. 2008;63: 8–160. 10.1111/j.1398-9995.2007.01620.x 18331513

[2] Wilson MS, Taylor MD, Balic A, Finney CA, Lamb JR, Maizels RM. Suppression of allergic airway inflammation by helminth-induced regulatory T cells. J Exp Med. 2005;202: 1199–1212. 10.1084/jem.20042572 16275759 PMC2213237

[3] Shi HZ, Qin XJ. CD4CD25 regulatory T lymphocytes in allergy and asthma. Allergy. 2005;60: 986–995. 10.1111/j.1398-9995.2005.00844.x 15969678

[4] Jaffar Z, Sivakuru T, Roberts K. CD4+CD25+ T cells regulate airway eosinophilic inflammation by modulating the Th2 cell phenotype. J Immunol. 2004;172: 3842–3849. 15004191 10.4049/jimmunol.172.6.3842

[5] Park HK, Cho KS, Park HY, Shin DH, Kim YK, Jung JS, et al Adipose-derived stromal cells inhibit allergic airway inflammation in mice. Stem Cells Dev. 2010;19: 1811–1818. 10.1089/scd.2009.0513 20225940

[6] Goodwin M, Sueblinvong V, Eisenhauer P, Ziats NP, LeClair L, Poynter ME, et al Bone marrow-derived mesenchymal stromal cells inhibit Th2-mediated allergic airways inflammation in mice. Stem Cells. 2011;29: 1137–1148. 10.1002/stem.656 21544902 PMC4201366

[7] Bonfield TL, Koloze M, Lennon DP, Zuchowski B, Yang SE, Caplan AI. Human mesenchymal stem cells suppress chronic airway inflammation in the murine ovalbumin asthma model. Am J Physiol Lung Cell Mol Physiol. 2010;299: L760–770. 10.1152/ajplung.00182.2009 20817776 PMC4116401

[8] Fu QL, Chow YY, Sun SJ, Zeng QX, Li HB, Shi JB, et al Mesenchymal stem cells derived from human induced pluripotent stem cells modulate T-cell phenotypes in allergic rhinitis. Allergy. 2012;67: 1215–1222. 10.1111/j.1398-9995.2012.02875.x. 22882409 PMC3555482

[9] Cho KS, Park HK, Park HY, Jung JS, Jeon SG, Kim YK, et al IFATS collection: Immunomodulatory effects of adipose tissue-derived stem cells in an allergic rhinitis mouse model. Stem Cells. 2009;27: 259–265. 10.1634/stemcells.2008-0283 18832595

[10] Sun YQ, Deng MX, He J, Zeng QX, Wen W, Wong DS, et al Human pluripotent stem cell-derived mesenchymal stem cells prevent allergic airway inflammation in mice. Stem Cells. 2012;30: 2692–2699. 10.1002/stem.1241 22987325 PMC3549478

[11] Cho KS, Roh HJ. Immunomodulatory effects of adipose-derived stem cells in airway allergic diseases. Curr Stem Cell Res Ther. 2010;5: 111–115. 19941459 10.2174/157488810791268681

[12] Cho KS, Park MK, Kang SA, Park HY, Hong SL, Park HK, et al Adipose-derived stem cells ameliorate allergic airway inflammation by inducing regulatory T cells in a mouse model of asthma. Mediators Inflamm. 2014;2014: 436476 10.1155/2014/436476 25246732 PMC4160627

[13] Ge X, Bai C, Yang J, Lou G, Li Q, Chen R. Intratracheal transplantation of bone marrow-derived mesenchymal stem cells reduced airway inflammation and up-regulated CD4^+^CD25^+^ regulatory T cells in asthmatic mouse. Cell Biol Int. 2013;37: 675–686. 10.1002/cbin.10084 23483727

[14] Fu QL, Chow YY, Sun SJ, Zeng QX, Li HB, Shi JB, et al Mesenchymal stem cells derived from human induced pluripotent stem cells modulate T-cell phenotypes in allergic rhinitis. Allergy. 2012;67: 1215–1222. 10.1111/j.1398-9995.2012.02875.x. 22882409 PMC3555482

[15] Nemeth K, Keane-Myers A, Brown JM, Metcalfe DD, Gorham JD, Bundoc VG, et al Bone marrow stromal cells use TGF-β to suppress allergic responses in a mouse model of ragweed-induced asthma. Proc Natl Acad Sci U S A. 2010;107: 5652–5657. 10.1073/pnas.0910720107 20231466 PMC2851758

[16] Ball HJ, Yuasa HJ, Austin CJ, Weiser S, Hunt NH. Indoleamine 2,3-dioxygenase-2: a new enzyme in the kynurenine pathway. Int J Biochem Cell Biol. 2009;41: 467–471. 10.1016/j.biocel.2008.01.005 18282734

[17] Baban B, Chandler PR, Sharma MD, Pihkala J, Koni PA, Munn DH, et al IDO activates regulatory T cells and blocks their conversion into Th17-like T cells. J Immunol. 2009;183: 2475–2483. 10.4049/jimmunol.0900986 19635913 PMC3677163

[18] Sharma MD, Baban B, Chandler P, Hou DY, Singh N, Yagita H, et al Plasmacytoid dendritic cells from mouse tumor-draining lymph nodes directly activate mature Tregs via indoleamine 2,3-dioxygenase. J Clin Invest. 2007;117: 2570–2582. 10.1172/JCI31911 17710230 PMC1940240

[19] Sucher R, Kurz K, Weiss G, Margreiter R, Fuchs D, Brandacher G. IDO-mediated tryptophan degradation in the pathogenesis of malignant tumor disease. Int J Tryptophan Res. 2010;3: 113–120. 22084593 10.4137/ijtr.s4157 PMC3195236

[20] Meisel R, Zibert A, Laryea M, Gobel U, Daubener W, Dilloo D. Human bone marrow stromal cells inhibit allogeneic T-cell responses by indoleamine 2,3-dioxygenase-mediated tryptophan degradation. Blood. 2004;103: 4619–21. 10.1182/blood-2003-11-3909 15001472

[21] Curti A, Trabanelli S, Salvestrini V, Baccarani M, Lemoli RM. The role of indoleamine 2,3-dioxygenase in the induction of immune tolerance: focus on hematology. Blood. 2009;113: 2394–2401. 10.1182/blood-2008-07-144485 19023117

[22] Ge W, Jiang J, Arp J, Liu W, Garcia B, Wang H. Regulatory T-cell generation and kidney allograft tolerance induced by mesenchymal stem cells associated with indoleamine 2,3-dioxygenase expression. Transplantation. 2010;90: 1312–1320. 10.1097/TP.0b013e3181fed001 21042238

[23] Luo C, Jia W, Wang K, Chi F, Gu Y, Yan X, et al Human amniotic fluid stem cells suppress PBMC proliferation through IDO and IL-10-dependent pathways. Curr Stem Cell Res Ther. 2014;9: 36–45. 24102581 10.2174/1574888x113086660067

[24] Ling W, Zhang J, Yuan Z, Ren G, Zhang L, Chen X, et al Mesenchymal stem cells use IDO to regulate immunity in tumor microenvironment. Cancer Res. 2014;74: 1576–1587. 10.1158/0008-5472.CAN-13-1656 24452999 PMC3959857

[25] Caterson EJ, Nesti LJ, Danielson KG, Tuan RS. Human marrow-derived mesenchymal progenitor cells: isolation, culture expansion, and analysis of differentiation. Mol Biotechnol. 2002;20: 245–256. 10.1385/MB:20:3:245 11936255

[26] Pellaton-Longaretti C, Boudousquie C, Barbier N, Barbey C, Argiroffo CB, Donati Y, et al CD4+CD25-mTGFbeta+ T cells induced by nasal application of ovalbumin transfer tolerance in a therapeutic model of asthma. Int Immunol. 2011;23: 17–27. 10.1093/intimm/dxq453 21123830

[27] Lee CG, Link H, Baluk P, Homer RJ, Chapoval S, Bhandari V, et al Vascular endothelial growth factor (VEGF) induces remodeling and enhances TH2-mediated sensitization and inflammation in the lung. Nat Med. 2004;10: 1095–1103. 10.1038/nm1105 15378055 PMC3434232

[28] Tournoy KG, Kips JC, Schou C, Pauwels RA. Airway eosinophilia is not a requirement for allergen-induced airway hyperresponsiveness. Clin Exp Allergy. 2000;30: 79–85. 10606934 10.1046/j.1365-2222.2000.00772.x

[29] Kang JH, Kim BS, Uhm TG, Lee SH, Lee GR, Park CS, et al Gamma-secretase inhibitor reduces allergic pulmonary inflammation by modulating Th1 and Th2 responses. Am J Respir Crit Care Med. 2009;179: 875–882. 10.1164/rccm.200806-893OC 19234107

[30] English K, Ryan JM, Tobin L, Murphy MJ, Barry FP, Mahon BP. Cell contact, prostaglandin E(2) and transforming growth factor beta 1 play non-redundant roles in human mesenchymal stem cell induction of CD4+CD25(High) forkhead box P3+ regulatory T cells. Clin Exp Immunol. 2009;156: 149–160. 10.1111/j.1365-2249.2009.03874.x 19210524 PMC2673753

[31] Mellor AL, Munn DH. IDO expression by dendritic cells: tolerance and tryptophan catabolism. Nat Rev Immunol. 2004;4: 762–764. 10.1038/nri1457 15459668

[32] Beutelspacher SC, Tan PH, McClure MO, Larkin DF, Lechler RI, George AJ. Expression of indoleamine 2,3-dioxygenase (IDO) by endothelial cells: implications for the control of alloresponses. Am J Transplant. 2006;6: 1320–1330. 10.1111/j.1600-6143.2006.01324.x 16686756

[33] Odemuyiwa SO, Ghahary A, Li Y, Puttagunta L, Lee JE, Musat-Marcu S, et al Cutting edge: human eosinophils regulate T cell subset selection through indoleamine 2,3-dioxygenase. J Immunol. 2004;173: 5909–5913. 15528322 10.4049/jimmunol.173.10.5909

[34] Uyttenhove C, Pilotte L, Theate I, Stroobant V, Colau D, Parmentier N, et al Evidence for a tumoral immune resistance mechanism based on tryptophan degradation by indoleamine 2,3-dioxygenase. Nat Med. 2003;9: 1269–1274. 10.1038/nm934 14502282

[35] Fallarino F, Grohmann U, You S, McGrath BC, Cavener DR, Vacca C, et al The combined effects of tryptophan starvation and tryptophan catabolites down-regulate T cell receptor zeta-chain and induce a regulatory phenotype in naïve T cells. J Immunol. 2006;176: 6752–6761. 16709834 10.4049/jimmunol.176.11.6752

[36] Munn DH, Mellor AL. Indoleamine 2,3-dioxygenase and tumor-induced tolerance. J Clin Invest. 2007;117: 1147–1154. 10.1172/JCI31178 17476344 PMC1857253

[37] Mbongue JC, Nicholas DA, Torrez TW, Kim NS, Firek AF, Langridge WH. The role of indoleamine 2,3-dioxygenase in immune suppression and autoimmunity. Vaccines. 2015;10: 703–729. 10.3390/vaccines3030703 PMC4586474 26378585

[38] Xu H, Oriss TB, Fei M, Henry AC, Melgert BN, Chen L, et al Indoleamine 2,3-dioxygenase in lung dendritic cells promotes Th2 responses and allergic inflammation. Proc Natl Acad Sci U S A. 2008;6: 6690–6695. 10.1073/pnas.0708809105 PMC2373348 18436652

[39] von Bubnoff D, Bieber T. The indoleamine 2,3-dioxygenase (IDO) pathway controls allergy. Allergy. 2012;67: 718–725. 10.1111/j.1398-9995.2012.02830.x 22519427

[40] Hayashi T, Beck L, Rossetto C, Gong X, Takikawa O, Takabayashi K, et al Inhibition of experimental asthma by indoleamine 2,3-dioxygenase. J Clin Invest. 2004;114: 270–279. 10.1172/JCI21275 15254594 PMC449749

[41] Moingeon P, Batard T, Fadel R, Frati F, Sieber J, Van Overtvelt L. Immune mechanisms of allergen-specific sublingual immunotherapy. Allergy. 2006;61: 151–165. 10.1111/j.1398-9995.2006.01002.x 16409190

[42] Taher YA, Piavaux BJ, Gras R, van Esch BC, Hofman GA, Bloksma N, et al Indoleamine 2,3-dioxygenase-dependent tryptophan metabolites contributes to tolerance induction during allergen immunotherapy in a mouse model. J Allergy Clin Immunol. 2008;121: 983–991. 10.1016/j.jaci.2007.11.021 18179817

[43] Croitoru-Lamoury J, Lamoury FM, Caristo M, Suzuki K, Walker D, Takikawa O, et al Interferon-γ regulates the proliferation and differentiation of mesenchymal stem cells via activation of indoleamine 2,3-dioxygenase (IDO). PLoS One. 2011;6: e14698 10.1371/journal.pone.0014698 21359206 PMC3040184

[44] Matysiak M, Stasiolek M, Orlowski W, Jurewicz A, Janczar S, Raine CS, et al Stem cells ameliorate EAE via an indoleamine 2,3-dioxygenase (IDO) mechanism. J Neuroimmunol. 2008;193: 12–23. 10.1016/j.jneuroim.2007.07.025 18077006 PMC2681256

[45] Opitz CA, Litzenburger UM, Lutz C, Lanz TV, Tritschler I, Koppel A, et al Toll-like receptor engagement enhances the immunosuppressive properties of human bone marrow-derived mesenchymal stem cells by inducing Indoleamine-2,3-dioxygenase-1 via interferon-beta and protein kinase R. Stem Cells. 2009;27: 909–919. 10.1002/stem.7 19353519

[46] Ebrahimi A, Kardar GA, Toolabi L, Ghanbari H, Sadroddiny E. Inducible expression of indoleamine 2,3-dioxygenase attenuates acute rejection of tissue-engineered lung allografts in rats. Gene. 2016;576: 412–420. 10.1016/j.gene.2015.10.054 26506443

[47] He Y, Zhou S, Liu H, Shen B, Zhao H, Peng K, et al Indoleamine 2,3-dioxygenase transfected mesenchymal stem cells induce kidney allograft tolerance by increasing the production and function of regulatory T cells. Transplantation. 2015;99: 1829–1838. 10.1097/TP.0000000000000856 26308414

[48] Lee YE, An J, Lee KH, Kim SS, Song HJ, Pyeon H, et al The synergistic local immunosuppressive effects of neural stem cells expressing indoleamine 2,3-dioxygenase (IDO) in an experimental autoimmune encephalomyelitis (EAE) animal model. PloS One. 2015;10: e0144298 10.1371/journal.pone.0144298 26636969 PMC4670164

[49] Ciccocioppo R, Cangemi GC, Kruzliak P, Gallia A, Betti E, Badulli C, et al Ex vivo immunosuppressive effects of mesenchymal stem cells on Crohn’s disease mucosal T cells are largely dependent on indoleamine 2,3-dioxygenase activity and cell-cell contact. Stem Cell Res Ther. 2015;6: 137 10.1186/s13287-015-0122-1 26206376 PMC4529692

[50] Fu J, Zhang L, Song S, Sheng K, Li Y, Li P, et al Effect of bone marrow-derived CD11b(+)F4/80 (+) immature dendritic cells on the balance between pro-inflammatory and anti-inflammatory cytokines in DBA/1 mice with collagen-induced arthritis. Inflamm Res. 2014;63: 357–367. 10.1007/s00011-014-0707-7 24458308

[51] Matysiak M, Stasiolek M, Orlowski W, Jurewicz A, Janczar S, Raine CS, et al Stem cells ameliorate EAE via an indoleamine 2,3-dioxygenase (IDO) mechanism. J Neuroimmunol. 2008;193: 12–23. 10.1016/j.jneuroim.2007.07.025 18077006 PMC2681256

[52] Krampera M, Cosmi L, Angeli R, Pasini A, Liotta F, Andreini A, et al Role for interferon-gamma in the immunomodulatory activity of human bone marrow mesenchymal stem cells. Stem Cells. 2006;24: 386–398. 10.1634/stemcells.2005-0008 16123384

[53] Spaggiari GM, Moretta L. Cellular and molecular interactions of mesenchymal stem cells in innate immunity. Immunol Cell Biol. 2013;91: 27–31. 10.1038/icb.2012.62 23146943

[54] Castro-Manrreza ME, Montesinos JJ. Immunoregulation by mesenchymal stem cells: biological aspects and clinical applications. J Immunol Res. 2015;2015: 394917 10.1155/2015/394917 25961059 PMC4417567

[55] Aggarwal S, Pittenger MF. Human mesenchymal stem cells modulate allogeneic immune cell responses. Blood. 2005;105: 1815–1822. 10.1182/blood-2004-04-1559 15494428

[56] Cui L, Yin S, Liu W, Li N, Zhang W, Cao Y. Expanded adipose-derived stem cells suppress mixed lymphocyte reaction by secretion of prostaglandin E2. Tissue Eng. 2007;13: 1185–1195. 10.1089/ten.2006.0315 17518704

[57] Cho KS, Lee JH, Park MK, Park HK, Yu HS, Roh HJ. Prostaglandin E2 and transforming growth factor- β play a critical role in suppression of allergic airway inflammation by adipose-derived stem cells. PLoS One. 2015;10: e0131813 10.1371/journal.pone.0131813 26176545 PMC4503681

[58] Chaves AC, Ceravolo IP, Gomes JA, Zani CL, Romanha AJ, Gazzinelli RT. IL-4 and IL-13 regulate the induction of indoleamine 2,3-dioxygenase activity and the control of Toxoplasma gondii replication in human fibroblasts activated with IFN-gamma. Eur J Immunol. 2001;31: 333–344. 10.1002/1521-4141(200102)31:2<333::AID-IMMU333>3.0.CO;2-X 11180096

