# Supplementary material for: Indoleamine 2,3-Dioxygenase Is Not a Pivotal Regulator Responsible for Suppressing Allergic Airway Inflammation through Adipose-Derived Stem Cells
Source: PLoS One. 2016 Nov 3;11(11):e0165661. doi: 10.1371/journal.pone.0165661 (PMC5094728; doi:10.1371/journal.pone.0165661)
Supplement: S1 Fig — ASCs show characteristics of mesenchymal stem cells in the immunophenotypic analysis (A), fibroblast-like morphology (B), adipogenesis (C), osteogenesis (D), chondrogenesis (E) (original magnification (PDF) [file pone.0165661.s001.pdf]

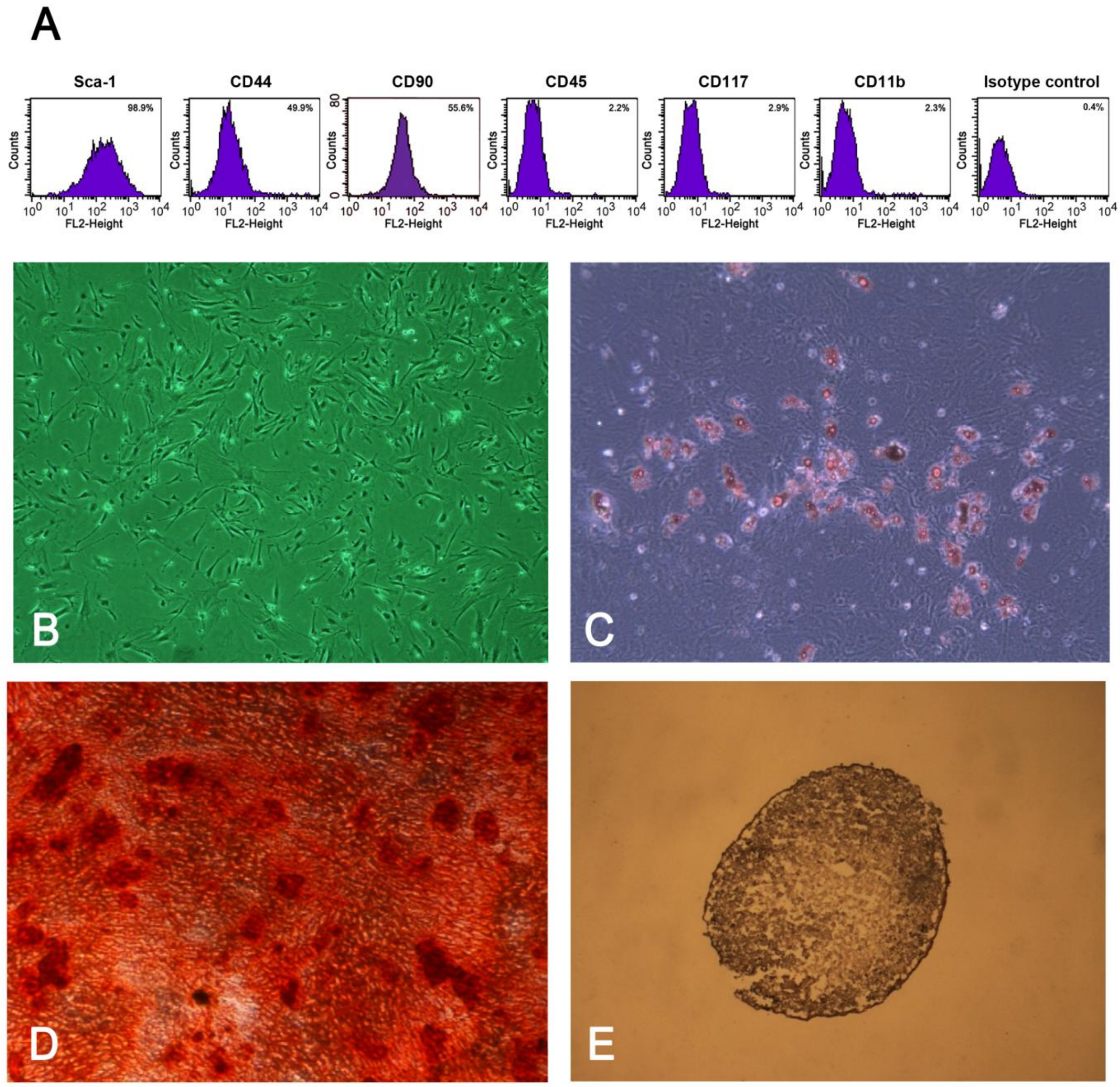

S1 Fig. Characteristics of adipose-derived stem cells (ASCs). ASCs show characteristics of mesenchymal stem cells in the immunophenotype (A), fibroblast-like morphology (B), and their capacity to differentiate toward the adipogenic (C), osteogenic (D), and chondrogenic (E) lineages (original magnification ×40).
